# Analyzing Cross-country Pandemic Connectedness During COVID-19 Using a Spatial-Temporal Database: Network Analysis

**DOI:** 10.2196/27317

**Published:** 2021-03-29

**Authors:** Amanda MY Chu, Jacky NL Chan, Jenny TY Tsang, Agnes Tiwari, Mike KP So

**Affiliations:** 1 Department of Social Sciences The Education University of Hong Kong Hong Kong China (Hong Kong); 2 Department of Information Systems, Business Statistics and Operations Management The Hong Kong University of Science and Technology Hong Kong China (Hong Kong); 3 Hong Kong Sanatorium & Hospital Hong Kong China (Hong Kong); 4 Li Ka Shing Faculty of Medicine The University of Hong Kong Hong Kong China (Hong Kong)

**Keywords:** air traffic, coronavirus, COVID-19, human mobility, network analysis, travel restrictions

## Abstract

Communicable diseases including COVID-19 pose a major threat to public health worldwide. To curb the spread of communicable diseases effectively, timely surveillance and prediction of the risk of pandemics are essential. The aim of this study is to analyze free and publicly available data to construct useful travel data records for network statistics other than common descriptive statistics. This study describes analytical findings of time-series plots and spatial-temporal maps to illustrate or visualize pandemic connectedness. We analyzed data retrieved from the web-based Collaborative Arrangement for the Prevention and Management of Public Health Events in Civil Aviation dashboard, which contains up-to-date and comprehensive meta-information on civil flights from 193 national governments in accordance with the airport, country, city, latitude, and the longitude of flight origin and the destination. We used the database to visualize pandemic connectedness through the workflow of travel data collection, network construction, data aggregation, travel statistics calculation, and visualization with time-series plots and spatial-temporal maps. We observed similar patterns in the time-series plots of worldwide daily flights from January to early-March of 2019 and 2020. A sharp reduction in the number of daily flights recorded in mid-March 2020 was likely related to large-scale air travel restrictions owing to the COVID-19 pandemic. The levels of connectedness between places are strong indicators of the risk of a pandemic. Since the initial reports of COVID-19 cases worldwide, a high network density and reciprocity in early-March 2020 served as early signals of the COVID-19 pandemic and were associated with the rapid increase in COVID-19 cases in mid-March 2020. The spatial-temporal map of connectedness in Europe on March 13, 2020, shows the highest level of connectedness among European countries, which reflected severe outbreaks of COVID-19 in late March and early April of 2020. As a quality control measure, we used the aggregated numbers of international flights from April to October 2020 to compare the number of international flights officially reported by the International Civil Aviation Organization with the data collected from the Collaborative Arrangement for the Prevention and Management of Public Health Events in Civil Aviation dashboard, and we observed high consistency between the 2 data sets. The flexible design of the database provides users access to network connectedness at different periods, places, and spatial levels through various network statistics calculation methods in accordance with their needs. The analysis can facilitate early recognition of the risk of a current communicable disease pandemic and newly emerging communicable diseases in the future.

## Introduction

Communicable diseases remain a major public health threat worldwide. The COVID-19 pandemic is a stark reminder of the ongoing challenge posed by communicable diseases on human health [1]. Timely surveillance and estimation of the risk of a pandemic are crucial for curbing the spread of communicable diseases. Without efficacious medications and vaccines, the implementation of nonpharmaceutical interventions, such as air travel restrictions and social distancing measures, is vital for controlling communicable diseases [2]. The conventional method of estimating the risk of a pandemic based only on the number of confirmed cases provides limited information about pandemic trends. Network analysis is a powerful tool to estimate the risk of a pandemic through network connectedness [3] when air travel is a common route of communicable disease transmission [4]. Network connectedness analysis using air travel data records can help visualize the effect of air travel restrictions on pandemic connectedness.

The risk of in-flight communicable disease transmission has been a global health concern well before the emergence of COVID-19 [5]. A number of in-flight communicable disease transmissions have been documented, including influenza [6], severe acute respiratory syndrome [7], multidrug-resistant tuberculosis [8], measles [9], meningococcal infections [10], norovirus [11], shigellosis [12], and cholera [13]. Studies on the transmission of influenza [14] and severe acute respiratory syndrome [15] on aircraft further indicated that air travel can serve as a channel for the rapid spread of newly emerging communicable diseases. A study on COVID-19 control in Latin America suggested that countries serving as air transportation hubs are more prone to disease transmission. The practicable use of travel data for the prediction of the risk of the COVID-19 pandemic has been previously reported [16].

We previously analyzed travel data retrieved from the web-based Collaborative Arrangement for the Prevention and Management of Public Health Events in Civil Aviation (CAPSCA) dashboard [17], which contains up-to-date and comprehensive meta-information on civil flights from 193 national governments in accordance with the airport, country, city, latitude, and the longitude of flight origin and the destination. Unlike official travel data sources, such as the Federal Aviation Administration (FAA) and the International Air Transport Association (IATA), the travel data of the CAPSCA are free and publicly available. The use of an Automated Dependent Surveillance Broadcast system as one of the travel data collection elements enables the CAPSCA to provide up-to-date travel locations, among other travel data (latitude and longitude of flight origin and the destination alongside a timestamp) [18,19]. The CAPSCA provides civil flight data for both passenger and cargo flights. While it is common knowledge that cargo flight crews may spread the disease through air travel, cargo is not always considered a health risk [20]. Travel data from both passenger and cargo flights are therefore more comprehensive for pandemic connectedness analysis.

Flexible analysis of travel data can be performed through in-database processing. Specifically, users can analyze travel data through various network statistics calculations, including network analysis [3], network density [21], and reciprocity [22], which are powerful tools to estimate the risk of a pandemic through network connectedness. Simple analyses with time-series plots and spatial-temporal maps would facilitate clear visualization of the analytical results. Time-series plots show changes in network density and reciprocity, which are likely to be early signs of alterations in the risk of a pandemic [21]. Spatial-temporal maps during network analysis illustrate the connectedness among places and reflect changes in the risk of a pandemic before unprocessed data on the number of confirmed cases are obtained [16]. Moreover, diverse tools can be used to analyze pandemic connectedness.

The CAPSCA dashboard allows us to create a spatial-temporal database by integrating the travel data from different airports to illustrate connectedness at the city, country, or regional levels in accordance with the users' preference. The database can facilitate research and policymaking at local and global levels and provide a spatial outlook of the evolution of the pandemic network for predicting and assessing the risk of a communicable disease pandemic.

## Methods

### Workflow of Data Collection and Analysis

**Figure 1:**
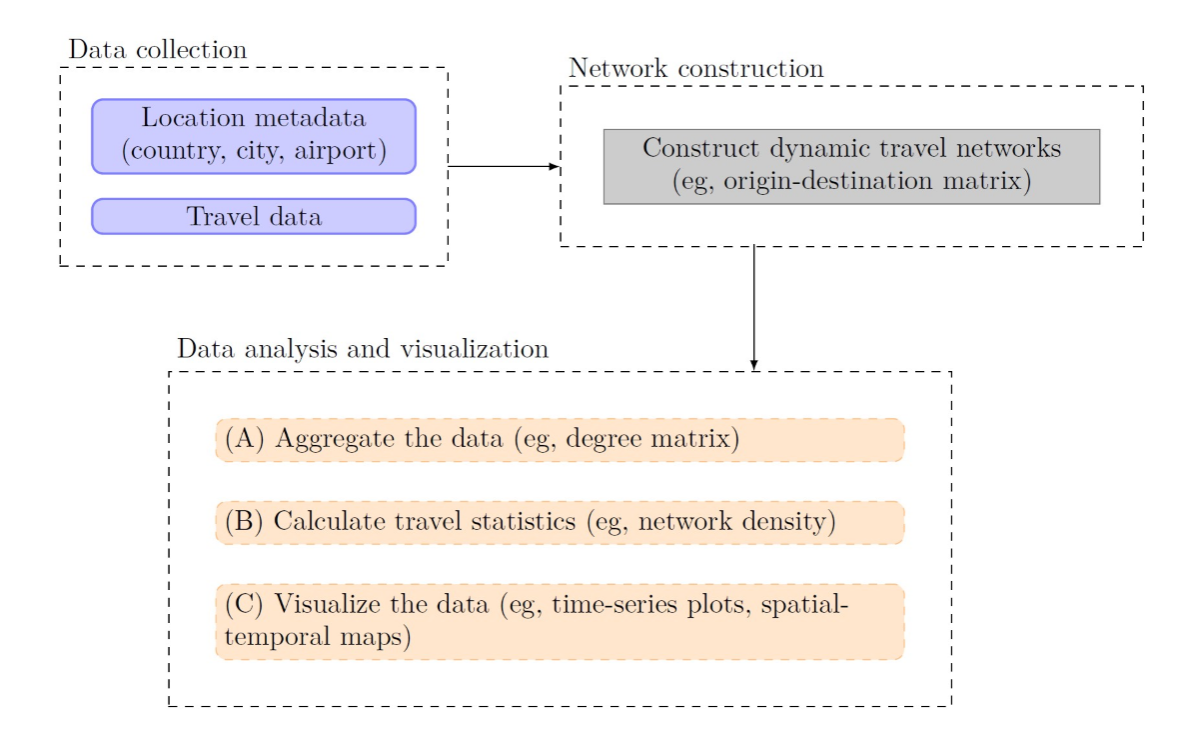
Workflow of data collection and analysis.

The database enables the visualization of pandemic connectedness through the workflow of travel data collection, network construction, data aggregation, travel statistics calculation, and visualization with time-series plots and spatial-temporal maps. The workflow of data collection and analysis is summarized in Figure 1.

### Data Collection

Travel data and meta-information were retrieved from the CAPSCA dashboard through two separate procedures:

1. Extracting the airport meta-information from the rendered JavaScript object “airportData” through a HyperText Transfer Protocol request.
2. Downloading and extracting the flight numbers of civil flights, including both passenger and cargo flights, from the JavaScript Object Notation responses through multiple Asynchronous JavaScript and Extensible Markup Language requests.

The collected travel data were filtered for valid International Civil Aviation Organization (ICAO)–formatted airport codes. Raw JavaScript Object Notation responses contain formatted airport codes from various data sources such as the ICAO, IATA, and FAA. Each type of formatted airport code has its specific format. Valid ICAO codes start only with a letter and have 4 letters or digits, valid IATA codes consist of 3 letters, while valid FAA codes are 3-5–character alphanumeric codes. Each format type helps develop a filtering system to extract travel data with specific codes.

### Data Records

A live version of the data record, which is maintained up-to-date with the latest data, can be downloaded from our travel database project repository [23]. The data records consist of 2 major parts: aggregated raw input and calculated or computed records.

The aggregated raw inputs are location metadata that contain data at multiple levels—country, city, airport, and geolocation (latitude and longitude)—and travel data, which contain daily information regarding flight origin and the destination, starting from January 2019. These data encompass >200 countries and regions worldwide.

The data records (details) are structured into the following 3 comma-separated value (CSV) files:

1. [ICAO_airport_meta.csv] Table of the location metadata (ICAO-CAPSCA airport meta). The fields of the table are as follows:
  - *countryName* is the name of the country
  - *countryCode* is the International Organization for Standardization (ISO)-3166 alpha-3 code of the country
  - *airportName* is the name of the airport
  - *airportCode* is the ICAO code of the airport
  - *cityName* is the name of the city
  - *latitude* is the geolocation (latitude) of the airport
  - *longitude* is the geolocation (longitude) of the airport
2. [flight_2019-01-01_2020-12-03.csv] Table of travel data (daily flight numbers from origin to destination). The fields of the table are as follows:
  - *date* is the record date
  - *num_flight* is the number of flights from the origin airport to the destination airport
  - *orig_airportCode* is the ICAO code of the origin airport
  - *orig_airportName* is the name of the origin airport
  - *orig_countryCode* is the ISO-3166 alpha-3 country code of the origin airport
  - *orig_countryName* is the country name of the origin airport
  - *orig_cityName* is the city name of the origin airport
  - *orig_latitude* is the geolocation (latitude) of the destination airport
  - *orig_longitude* is the geolocation (longitude) of the destination airport
  - *dest_airportCode* is the ICAO code of the destination airport
  - *dest_airportName* is the name of the destination airport
  - *dest_countryCode* is the ISO-3166 alpha-3 country code of the destination airport
  - *dest_countryName* is the name of the country where the destination airport is located
  - *dest_cityName* is the name of the city were the destination airport is located
  - *dest_latitude* is the geolocation (latitude) of the destination airport
  - *dest_longitude* is the geolocation (longitude) of the destination airport
3. [network_statistics.csv] Table of the calculated network statistics. The fields of the table are as follows:
  - *date* is the reference date of the network statistics at time t
  - *V_t_* is the number of vertices (Vt) at time t
  - *E_t_* is the number of edges (Et) at time t
  - *D_t_* is the edge density (Dt) at time t
  - *R_t_* is the reciprocity (Rt) at time t

### Dynamic Network Construction and Data Aggregation

The travel data can be used to construct the travel network structure [24]. The basic network components involve nodes (vertices) and links (edges). The nodes represent the target entity (location), such as airport, city, or country. As the travel data contain detailed airport-to-airport records, they can be transformed by merging data from the airports to form nodes of cities, countries, regions, or groups of any geolocations in accordance with the users’ preference. A link represents a relationship (connection) between 2 target entities. The relationship can be binary or numeric (eg, flight frequency), indicating the existence or strength of a travel connection, respectively.

For example, if we focus on global analysis, we aggregate the airport data at the country level and input the country data as a new set of nodes to form travel subnetworks, which are represented by the country-country–origin-destination matrix with entries being the flight frequencies between 2 countries.

### Data Analysis: Travel Network Statistics

**Figure 2:**
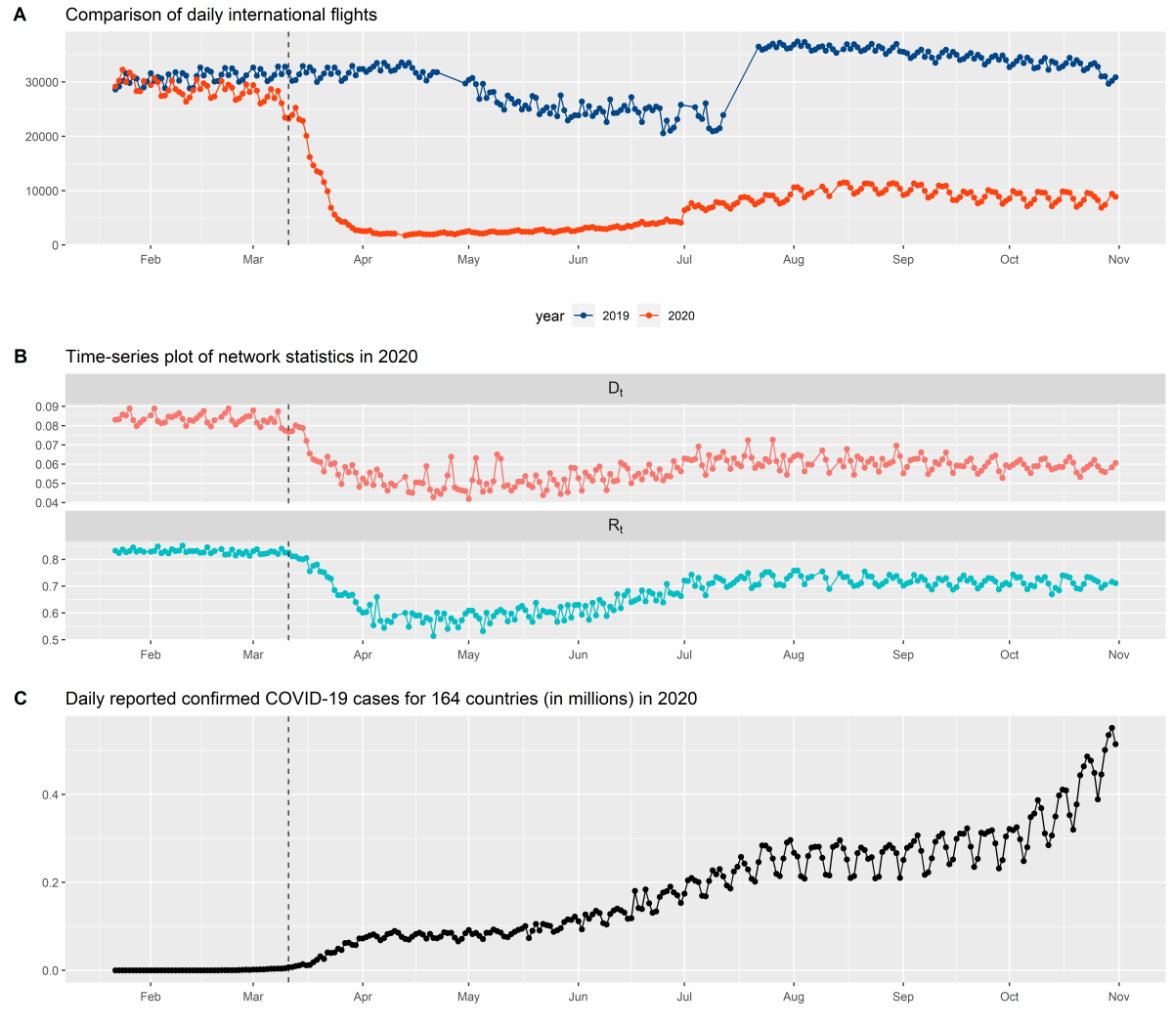
Time-series plots of (A) daily international flights, (B) global network statistics, and (C) daily reported number of confirmed COVID-19 cases.

**Figure 3:**
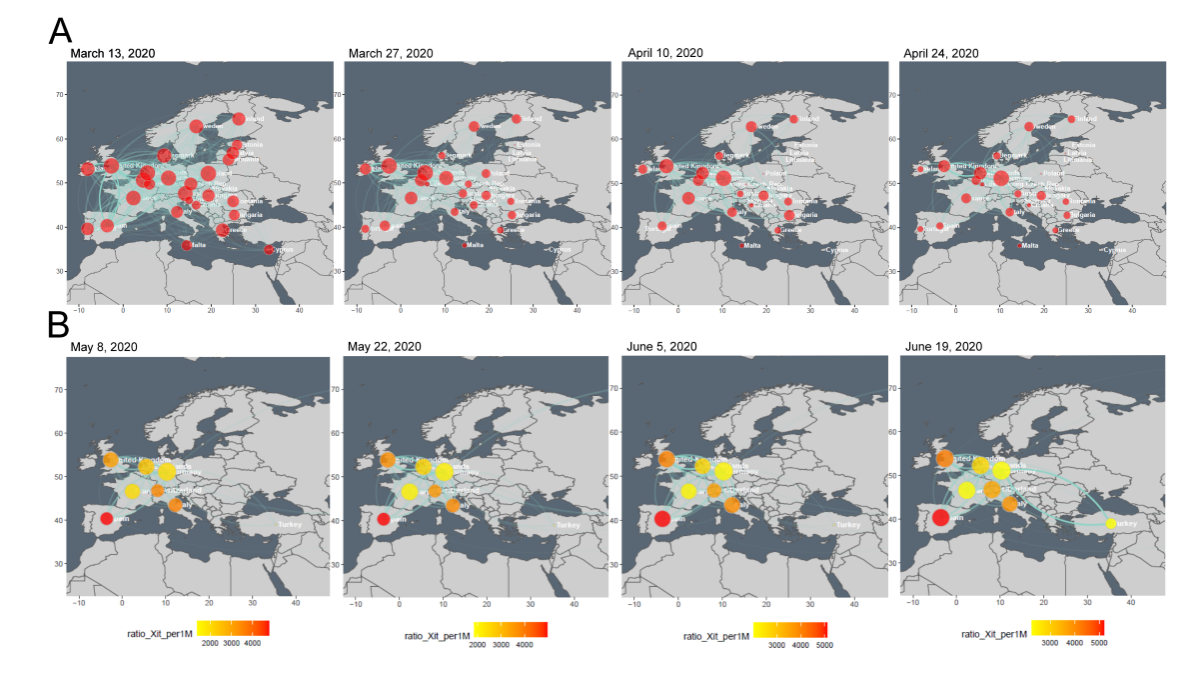
Spatial-temporal maps of connectedness in Europe at (A) the country level between March 13 and April 24, 2020, and (B) air transportation hub level between May 8 and June 19, 2020.

We can further aggregate the travel data to obtain overall worldwide flight information. Figure 2A shows the time-series plots of worldwide daily flights in 2019 and 2020. By comparing the 2 time-series curves in Figure 2A, we observed similar patterns of worldwide daily flights from January to early-March in 2019 and 2020. A sharp decline in the number of daily flights recorded in mid-March 2020 was likely related to large-scale air travel restrictions owing to the COVID-19 pandemic. In addition, the subnetwork can be further used to generate the degree matrix, where its diagonal entries contain the number of edges connected to different nodes (number of connected countries of each node). The combined use of the origin-destination and degree matrices can yield spatial-temporal maps (Figure 3).

If V_t_ is the number of vertices of the dynamic network at time t, and E_t_ is the number of edges of the dynamic network at time t, network statistics such as D_t_ (network density [21]) and Rt (reciprocity [22]) can be determined (Figure 2A). For example, in the dynamic networks in Figure 3, countries are represented by vertices, and travel connections are represented by edges. Network density D_t_ is based on an undirected network structure, and is defined as follows:



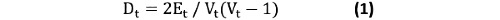



which refers to the ratio of the number of connections with respect to the maximum possible connections among countries. This equation illustrates how dense the connections in the dynamic network are at time *t*.

Reciprocity R_t_ is based on a directed network structure and is defined as follows:



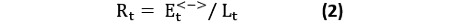



where R_t_ is the ratio of the number of links pointing in both directions, E_t_^<->^ (mutual links), to the total number of links, L_t_. In other words, the value of R_t_ represents the average possibility that a link is reciprocated.

## Results

### Data Visualization

Network statistics data were visualized using time-series plots. In addition, data records of the origin-destination and degree matrices can be visualized using spatial-temporal maps.

Figure 2 displays the time-series plots of worldwide daily flights (Figure 2A), global network statistics (Figure 2B), and daily reported confirmed COVID-19 cases (Figure 2C). We found that changes in network density can serve as an early signal of the risk of a pandemic.

The time-series plots of network density and reciprocity shown in Figure 2B demonstrate the practical use of network statistics to predict the risk of a global pandemic. With initial reports of COVID-19 cases worldwide, a high network density and reciprocity in early-March 2020 were early signals of the COVID-19 pandemic and were associated with the rapid increase in COVID-19 cases reported by the World Health Organization (WHO) in mid-March 2020 (Figure 2C). The sharp reduction in the network density and reciprocity in mid-March 2020 suggests a reduction in the risk of the pandemic, which was associated with a steady number of daily confirmed cases from mid-March to May 2020. The gradual increase in network density and reciprocity from mid-May 2020 suggests an increase in the pandemic risk, which was associated with a rapid surge in COVID-19 cases from mid-May to August 2020. The in-database processing system allows users to analyze network density and reciprocity at local or global levels during selected periods in order to identify possible trends in the evolution of the risk of pandemics.

Figure 3 shows some spatial-temporal maps to illustrate the network connectedness among countries under different spatial levels. The levels of connectedness among places are strong indicators of the risk of a pandemic. The spatial-temporal maps showing the connectedness among places are constructed on the basis of the origin-destination and degree matrices, which provide directional data and summarized node data (degree of vertices), respectively. The maps display the connectedness among places in the form of connections and bubbles. The connections (links) illustrate the connectedness among places. The bubbles (vertices), which contain aggregated information presented by the node size, further enhance the visualization of connectedness, especially when the network density is high. The in-database processing system allows users to generate spatial-temporal maps at multiple spatial levels, such as airports, cities, countries, or any regions of interest, with various temporal settings, such as different periods and time-intervals. The flexibility of the database facilitates data analysis in accordance with the users’ preferences.

For example, to analyze the connectedness in Europe during the first wave of the COVID-19 pandemic in early 2020, spatial-temporal maps of Europe at 14-day intervals between March and April 2020 were generated (Figure 3A). Maps displaying connectedness at the country level are plotted, with each bubble representing a country. The size of the colored bubbles represents the number of vertices or countries. The color intensity of the bubbles indicates the number of daily confirmed COVID-19 cases per 1 million population of the country. The light-green connections (links) represent the number of daily international flights. Thicker the green line, higher the connectedness between 2 countries. The map for March 13, 2020, shows the highest level of connectedness among European countries, reflecting a severe outbreak of COVID-19 in late March and early April 2020. The significant reduction in connectedness from March 13 to April 24 indicates that the first wave of the pandemic subsided in May 2020.

Figure 3B shows a different set of spatial-temporal maps to analyze connectedness in Europe before the onset of the second wave of the COVID-19 pandemic. Spatial-temporal maps of Europe at 14-day intervals between May and June 2020 were generated. As countries serving as air transportation hubs were particularly prone to the spread of COVID-19 [16], spatial-temporal maps at the country level for these hubs have been plotted to investigate network connectedness between the countries harboring these hubs during the growth of the pandemic. Each bubble represents a country with airports defined as among the top 10 air transportation hubs in Europe by the Official Airline Guide MegaHub Index of 2019 [25]. The color indicates the accumulated total number of confirmed COVID-19 cases per 1 million population of a particular country at time *t* (Figure 3B, ratio_Xit_per1M). The significant increase in connectedness in mid-June 2020 indicates an increase in the risk of the pandemic in Europe, especially among hub countries.

The aforementioned data were visualized using igraph in the R software package (The R Foundation) or software facilitating network visualization, such as Gephi (The Gephi Consortium).

### Technical Validation

CAPSCA is a voluntary cross-sectorial and multi-organizational collaborative program managed by the ICAO with support from the WHO. The quality of the data on the dashboard should be guaranteed. As a quality control measure, we used the aggregated number of international flights from April to October 2020 to compare the number of COVID-19 cases at hub airports officially reported by the ICAO [26] with the data collected from the CAPSCA dashboard. We analyzed the correlation between the 2 data sets for 7 different regions during this period: Asia Pacific, East and South Africa, Europe and North Atlantic, Middle East, North America and the Caribbean, South America, and West and Central Africa. The results are summarized in Table 1. We found that most coefficients of correlation were >0.99, except for the Asia Pacific region (r=0.96). This difference probably results from data synchronization (lagging by 3 days) by the ICAO after collecting the daily raw counts.

**Table 1:** Correlation between the number of daily international flights officially reported by the International Civil Aviation Organization and those obtained from the Collaborative Arrangement for the Prevention and Management of Public Health Events in Civil Aviation dashboard for 7 geographic regions from April to October 2020.

ICAO^a^-identified geographic regions	Coefficient of correlation
Asia Pacific	0.96
East and South Africa	1.00
Europe and North Atlantic	1.00
Middle East	1.00
North America and the Caribbean	0.99
South America	1.00
West and Central Africa	1.00

^a^ICAO: International Civil Aviation Organization.

## Discussion

### Principal Findings

Ongoing systematic surveillance is important to help detect early outbreaks and to evaluate the effectiveness of public health measures and programs [27,28]. In this study, we attempted to conduct flexible analysis of freely available travel data collected from the CAPSCA dashboard to identify certain patterns and early signals of the COVID-19 pandemic, which may help policymakers take appropriate action. By linking the database to daily numbers of confirmed COVID-19 cases, we can develop a user-friendly platform for timely and flexible visualization of network connectedness to facilitate surveillance and early recognition of the risk of a pandemic, including the high network density and reciprocity in early March 2020 through time-series analysis and the high level of connectedness among European countries on March 13, 2020, through spatial-temporal mapping. The surveillance and findings are important for curbing the spread of communicable diseases and balancing disease control and economic recovery.

Our study provides a clear workflow for data collection and analysis and the suggested software for analysis. Because air transportation is highly relevant to the dissemination of communicable diseases, the database and the analysis can also be applied to investigate the risk of other communicable disease pandemics occurring currently or emerging in the future. In addition, researchers may replicate our workflow for assessing pandemic connectedness by using the same database or other databases.

For data quality assurance, we conducted correlation analysis and validated the database. We compared the number of aggregated international flights officially reported by the ICAO for 7 regions from April to October 2020 with corresponding data we collected from the CAPSCA dashboard. We found only minor differences probably owing to data synchronization performed by the ICAO after collecting the daily raw counts of COVID-19 cases. We expect that our findings may help researchers explore and validate freely available health-related databases to conduct ongoing and systematic analysis and interpretation to identify early warning signals, such that necessary action can be taken to prevent and control the spread of communicable diseases.

### Conclusions

In this study, we demonstrated a workflow to analyze freely available travel data retrieved from the CAPSCA dashboard, together with data on confirmed COVID-19 cases reported by the WHO, for systematic surveillance. Flexible analysis of the travel data can be performed through in-database processing, which allows us to visualize and analyze the risk of a pandemic and pandemic connectedness by using different tools such as time-series plots and spatial-temporal maps. This analysis facilitates early recognition of the risk of pandemics of current communicable diseases and newly emerged communicable diseases in the future.

## Abbreviations

CAPSCA: Collaborative Arrangement for the Prevention and Management of Public Health Events in Civil Aviation
CSV: comma-separated value
FAA: Federal Aviation Administration
IATA: International Air Transport Association
ICAO: International Civil Aviation Organization
ISO: International Organization for Standardization
WHO: World Health Organization
